# An innovative total temporomandibular joint prosthesis with customized design and 3D printing additive fabrication: a prospective clinical study

**DOI:** 10.1186/s12967-018-1759-1

**Published:** 2019-01-03

**Authors:** JiSi Zheng, XuZhuo Chen, WenBo Jiang, ShanYong Zhang, MinJie Chen, Chi Yang

**Affiliations:** 1 Department of Oral Surgery, Ninth People’s Hospital, College of Stomatology, Shanghai Jiao Tong University School of Medicine, Shanghai Key Laboratory of Stomatology and Shanghai Research Institute of Stomatology, No. 639 Zhi Zao Ju Rd, Shanghai, 200011 China; 2 Center of 3D-printing Translational Medicine, Ninth People’s Hospital, Shanghai Jiao Tong University School of Medicine, Shanghai, China

**Keywords:** Temporomandibular joint, Customized prosthesis, 3D printing, Clinical application

## Abstract

**Background:**

Total temporomandibular joint (TMJ) prosthesis is an effective and reliable method of joint reconstruction. However, there is still an urgent need to design a new TMJ prosthesis because of no commercially available TMJ prosthesis appropriate for the clinical application on the Chinese population. This study was introduced to prospectively confirm the safety and effectiveness of a new TMJ prosthesis with customized design and 3D printing additive fabrication in clinical application.

**Methods:**

Patients with unilateral end-stage TMJ osteoarthrosis were recruited in this study from Nov 2016 to Mar 2017. Computed tomography scans for all patients were obtained and transformed into three-dimensional (3D) reconstruction models. The customized TMJ prosthesis consisted of three components including the fossa, condylar head, and mandibular handle units, which were designed based on the anatomy of the TMJ and were fabricated using the 3D printing technology. The prominent characters of the prosthesis were the customized design of the fossa component with a single ultra-high-molecular-weight polyethylene and the connection mechanism between the condylar head (Co–Cr–Mo alloy) and mandibular handle components (Ti6Al4 V alloy). The clinical follow-up, radiographic evaluation and laboratory indices were all done to analyze the prosthesis’ outcomes in the clinical application.

**Results:**

12 consecutive patients were included in the study. There were no complications (infection of the surgical wound, damage of liver and kidney, displacement, breakage, or loosening of the prosthesis) found after surgery. Pain, diet, mandibular function, and maximal interincisal opening showed significant improvements after surgery. But the lateral movement was limited to the non-operated side and the mandible deviated towards the operated side on opening mouth following surgery.

**Conclusions:**

The presented TMJ prosthesis is considered an innovative product in TMJ Yang’s system, which is unique compared to other prostheses for the special design and 3D printing additive manufacture. Moreover, the prosthesis is very safe and efficient for clinical use.

*Trial registration* Prospective reports on Chinese customized total temporomandibular joint prosthesis reconstruction cases, ChiCTR-ONC-16009712. Registered 22 Nov 2016, http://www.chictr.org.cn/showproj.aspx?proj=16091

## Background

Temporomandibular joint (TMJ) is often affected by a wide spectrum of disorders, including extra-articular and intra-articular pathologies which usually present with various clinical symptoms including pain in the preauricular region, limitation of mouth opening, malocclusion, or jaw deformity [1, 2]. The former is typically managed non-surgically, whereas the latter is often managed surgically. Some of the intra-articular TMJ diseases, including end-staged TMJ osteoarthritis, severe idiopathic condylar resorption, TMJ ankylosis, comminuted condylar fracture, and part of TMJ tumors, have to be treated by simultaneously removing the lesion and joint together, with primary joint reconstruction to restore its anatomic structure and function as much as possible [3–5].

**Fig. 1:**
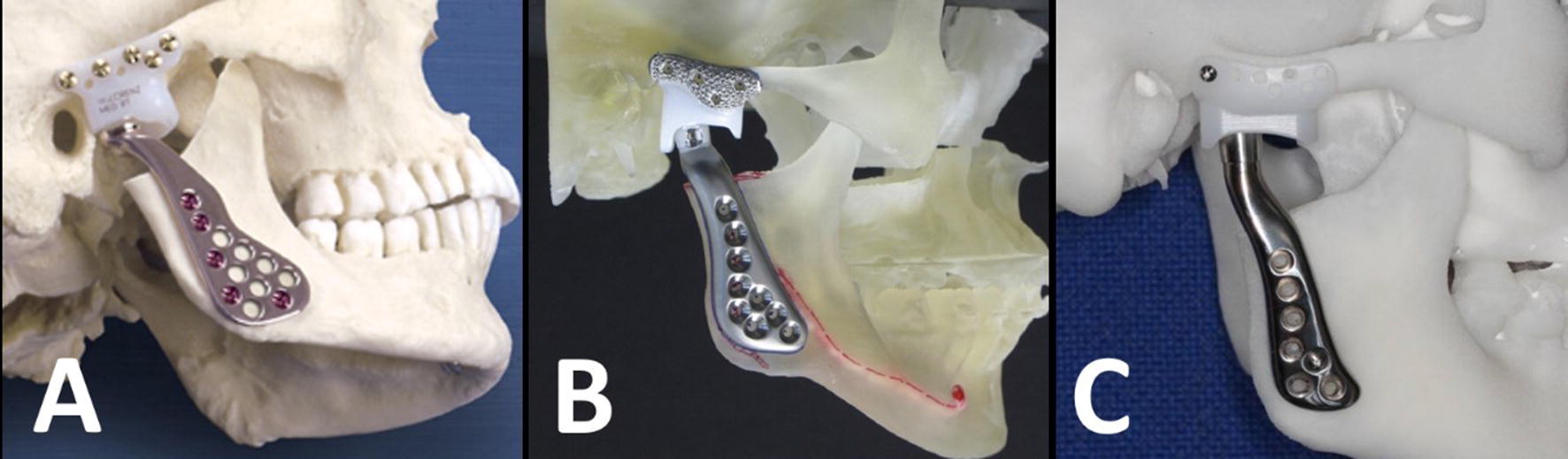
The comparison between the new prosthesis and the Zimmer Biomet or TMJ concepts products. **a** The Zimmer Biomet prosthesis. **b** The TMJ concepts prosthesis. **c** The TMJ prosthesis in this study

Total alloplastic TMJ prosthesis is one of the effective and efficient methods of joint reconstruction [3, 5]. With the development of hip joint prosthesis for the treatment of severe hip lesions, first reported by John Charney in 1961 [6, 7], TMJ prosthesis has also been gradually applied in the field of craniomaxillofacial surgery with various rates of successes [8, 9]. Up till now, there are two main commercially available TMJ prostheses for clinical applications: the stock or custom-made Zimmer Biomet products (Biomet microfixation, Jacksonville, FL, USA) [10] and the custom-made TMJ Concepts product (TMJ Concepts Inc, Camarillo, CA, USA) [11] (Fig. 1a, b). Unfortunately, there are no registration certificates available for the customized Zimmer Biomet and TMJ Concepts prostheses in China, in addition to their higher cost (20,000 USD per joint), which is far beyond the affordability of most Chinese populations [11]. Although, the stock Zimmer Biomet has a registration certificate for clinical use in China, and it is much cheaper than the customized products, however, it does not always match the Chinese patient’s TMJ anatomy very well [12–14]. For these reasons, the TMJ prosthesis as a better clinical solution cannot be satisfactorily applied to the Chinese patients [15]. Accordingly, various autogenous tissues, encountering the risks of a second operation for donor site, including the costochondral graft [16] or sternoclavicular joint graft [17], are frequently harvested as an alternative to the artificial prosthesis in China to replace the severely diseased joint.

Undoubtedly, there is a great need for the research and development of Chinese TMJ prosthesis in domestic TMJ surgery. Based on previous experiences, the authors have designed a newly customized TMJ prosthesis (Fig. 1c), which is completely different from the Zimmer Biomet and TMJ Concepts [18]. Moreover, the combined application of three-dimensional (3D) Printing and computer-aided design and computer-aided manufacturing (CAD/CAM) technologies plays a crucial role in manufacturing this prosthesis. In addition, the biomechanical and biological properties tests, including the fatigue resistance test, functional load capacities, wear testing, and animal experiments, have been all conducted by School of Materials Science and Engineering in Shanghai Jiao Tong University, in accordance to the testing methods accomplished by the Zimmer Biomet and TMJ concepts [18–20], and the results proved that this prosthesis functions well both in vitro and in vivo. The purpose of this study was to evaluate the safety and efficacy of the new TMJ prosthesis with customized design and 3D printing additive manufacturing in clinical application.

## Methods

### Patients

This was a prospective clinical study conducted at the Department of Oral Surgery, Shanghai Ninth People’s Hospital between Nov 2016 and Mar 2017. In this study, patients were recruited for TMJ reconstruction with a new prosthesis based on the indications and contraindications as follows.

### Inclusion criteria

- Unilateral end-stage TMJ osteoarthrosis (stage V of Wilkes-Bronstein Classification) with a stable occlusion relationship [21].

### Exclusion criteria

- Allergy to prosthetic components materials.
- Uncontrollable masticatory muscle hyperfunction or parafunctional habits (clenching or grinding).
- Active or even suspected infections in or around the implantation site of the prosthesis.
- History of previous TMJ surgeries.
- Systemic diseases contraindicating the use of the artificial prosthesis [22–24].

This study was approved by Shanghai Ninth People’s Hospital Human Research Ethics Committee. Moreover, the principles outlined in the Declaration of Helsinki were followed in the study as well. All patients were informed about the surgical purpose, management protocol, recovery period, and possible complications. An informed consent was obtained from all participants.

### TMJ prosthesis preparation prior to surgery

**Fig. 2:**
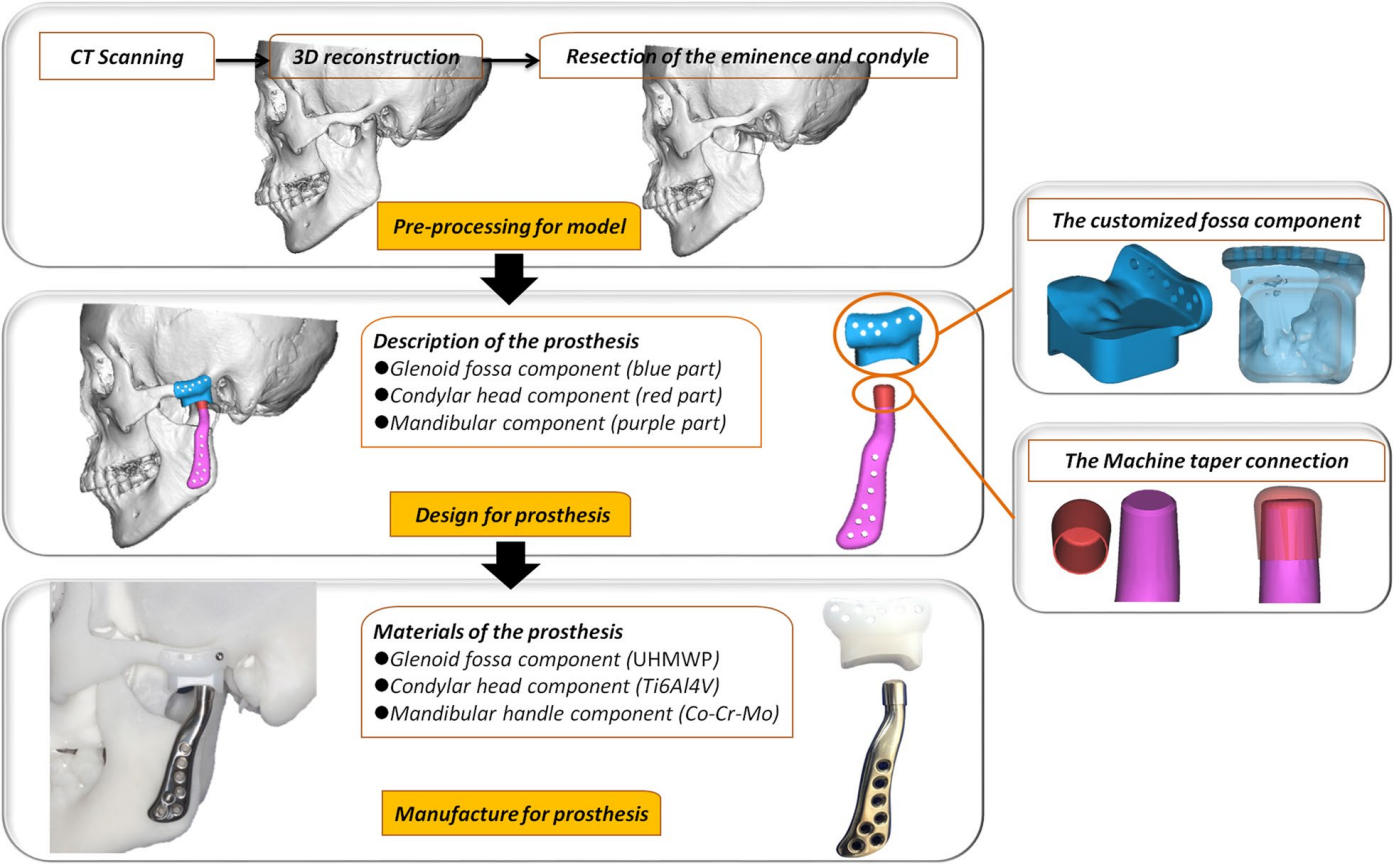
The processing of the new TMJ prosthesis, including the pre-processing for the craniomaxillofacial model, the design for the prosthesis, and the manufacture for the prosthesis. The main innovative points of the prosthesis are the customized fossa component with single UHMWP and the Machine tape mechanism for the connection between the condylar head (Co–Cr–Mo alloy) and mandibular handle components (Ti6Al4 V alloy)

- CT scan (GE Heal-thcare, Buckinghamshire, England) of the entire mandible, maxilla, and TMJ for all patients (0.625 mm slice thickness).
- Processing CT data with DICOM format to create the 3D craniomaxillofacial model in Mimics software 18.0 (Materialize Co, Leuven, Belgium).
- Cutting the lower part of the eminence and entire condyle with the aid of Mimics software.
- Designing the prosthesis (including the glenoid fossa, condylar head, and mandibular handle components) by using 3-Matic research software 9.0 (Materialize Co, Leuven, Belgium). The main principles of the TMJ prosthesis design are focusing on the following points: (1) the dimension and slope of the articular surface of the fossa component are ascertained based on the Chinese TMJ anatomy database in our previous study [25]; (2) the bony surface of the fossa part is customized to match the anatomic configuration of the glenoid fossa, zygomatic arch, and remaining articular eminence; (3) the condylar head component is cylinder-like shaped with a hollow structure, which is perfectly fitted in with the predefined cone frustum on the top of the mandibular handle component according to the machine taper connection mechanism [26, 27]; (4) the inner surface of the handle component is also customized to fit with the external surface of the mandibular ramus [23].
- Manufacturing the three components of the prosthesis: The fossa component is fabricated from ultra-high-molecular-weight polyethylene (UHMWP, GB/T19701.2) by 5-axis milling device (DMU60, DGM, Germany). The condylar head component is fabricated from the cobalt-chromium-molybdenum alloy (Co–Cr–Mo alloy, YY0117.3) by 5-axis milling device. The mandibular component is fabricated from titanium alloy (Ti6Al4 V alloy, GB/T13810) by a 3D-printing machine (Arcam A1, MÖlnda, Sweden). Then, all of the components are polished and the medial surface of the mandibular handle is treated with the sandblasting technique.
- Fitting the prosthesis in the 3D skull model before sterilizing and packaging: The three components of the prosthesis are fitted in the 3D model to check whether the stability and accuracy of each individual component are the same with the models in Mimics software.
- Sterilization and packaging of the prosthesis: All TMJ prosthesis components are provided clean and non-sterile and therefore, no additional cleaning prior to sterilization is needed. The glenoid fossa component is sterilized utilizing ethylene oxide gas sterilization, and the condylar head and mandibular components are sterilized using steaming sterilization. Afterward, The TMJ prosthesis components are repackaged again. The simple processing procedure is showed in Fig. 2.

### Surgical procedure

**Fig. 3:**
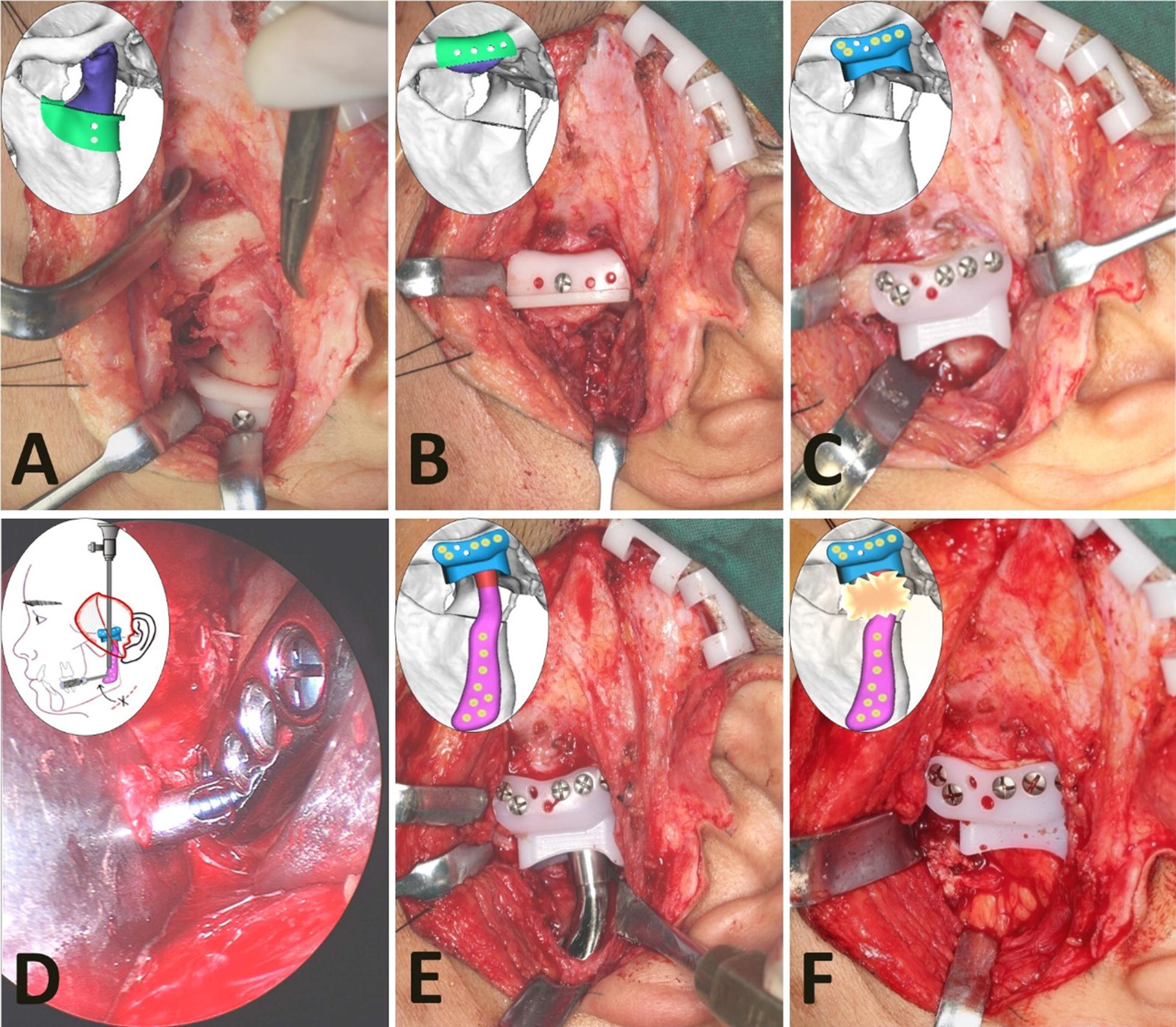
The surgical procedure for the new TMJ prosthesis. **a**, **b** The resection of the entire condyle and the lower part of the articular eminence by using the surgical templates. **c** The fixation of the fossa component with the guide of the holes in the template. **d**, **e** The fixation of the mandibular handle component with the help of the endoscope and transbuccal retractor. **f** The graft of free fat harvested from the buccal fat pad to fill into the space around the prosthesis neck
- The fossa components were fixed with titanium screws firstly (Stryker Fixation System, Kalamazoo, USA) based on the orientation holes of the templates (Fig. 3c).
- The mandibular handle with condylar head components facilitated the insertion of the titanium screws with the previous holes in the template. Then, the lower resultant holes of the mandibular component were implanted with the screws with the help of the endoscope (accessed through the preauricular incision) and transbuccal retractor (inserted through a 3 mm incision in parotideo-masseteric region) after the occlusion was guaranteed as stable as preoperatively (Fig. 3d, e).
- A piece of fat graft was harvested from the buccal fat pad and then placed around the condylar head component to obliterate the resultant space and to prevent the formation of heterotopic bone around the prosthesis (Fig. 3f) [13, 28, 29].
- The occlusion was checked again, and the wound was closed in layers with an 18-gauge drain.

- All patients received general anesthesia through nasal intubation.
- A modified preauricular approach was performed in all patients to expose the zygomatic arch, eminence, condyle, and lateral mandibular ramus.
- The entire condyle (Fig. 3a) and the lower part of the articular eminence (Fig. 3b) were osteotomized guided by the surgical templates with the orientation holes and planes [14].

### Evaluation of clinical safety

The clinical parameters and laboratory investigations were used to evaluate the clinical safety.

#### Clinical general check-ups

The maxillofacial general check-ups included (a) infection, (b) dental malocclusion, and (c) incision healing in 1 week, 1, 3, 6, and 12 months postoperatively.

#### Radiographic examinations

The displacement, breakage, or loosening of the prosthesis components were checked in CT scans at 1 week and 12 months postoperatively [13, 14].

#### Laboratory investigations

The laboratory indices included (a) routine blood tests; (b) kidney function tests; (c) liver function tests; (d) routine urine tests; and (e) routine stool tests. These tests were performed and recorded 1 week preoperatively, 1, and 12 months postoperatively.

### Evaluation of clinical efficacy

The subjective and objective indices were used to assess the clinical efficacy. These data were collected using a standardized data collection format 1 week preoperatively, 1, 3, 6, and 12 months postoperatively. Quantitative measurements were performed by two oral and maxillofacial surgeons together. When there was a disagreement, the consensus was reached by a discussion.

#### Subjective assessment indices

Subjective data including (a) pain, (b) functions of the mandible, and (c) diet, were obtained using a 10-length visual analog scale (VAS). The pain scale ranged from no pain at 0 to worst pain at 10. The mandibular functions scale ranged from no loss at 0 to complete loss of functions at 10. The diet scale ranged from no restriction at 0 to only liquids at 10 [22–24].

#### Objective assessment indices

Objective measurements of the mandibular range of motion, including (a) maximal interincisal opening, (b) lateral movements (left and right), (c) forward movement, and d) mandibular deviation when opening the mouth, were recorded in millimeters. (MIO means maximal interincisal opening, MDS means movement towards the diseased (operated) side; MNS means movement to normal (non-operated) side, MFM means mandible forward movement, MOD means mouth opening deviation to the diseased side) [22, 23].

### Statistical analysis

Data were analyzed using the Statistical Package for Social Sciences software package, version 17.0 (SPSS, Chicago, IL). The subjective and objective assessment indices before and after surgery were compared using the paired t-test of one-way analysis of variance. A P value of less than 0.05 was considered statistically significant (* is P ≤ 0.05, ** is P ≤ 0.01, *** is P ≤ 0.001, **** is P ≤ 0.0001).

## Results

### Patients data

12 consecutive patients were included in the study based on the inclusion and exclusion criteria. There were 7 females and 5 males. Their mean age was 47.8 years (range, 35 to 66 years), and the mean duration of the disease was 4.9 years (range, 0.5 to 15 years). The left side was affected in 5 patients and the right side in 7 (Table 1). All patients were treated primarily with the conservative therapy for an average of 2.37 months (range, 0.4 to 6 months) with no obvious clinical improvements.

**Table 1:** Basic data of the unilateral end-stage TMJ osteoarthrosis patients treated by the new TMJ prosthesis

No.	Sex	Age (years)	Side	Duration (years)	Consecutive therapy (years)
1	M	65	R	2	1.5
2	F	54	L	2	1.5
3	M	53	L	0.75	0.5
4	F	40	R	10	2
5	F	38	R	15	2
6	F	46	L	3	2.5
7	M	35	L	0.5	0.4
8	F	66	R	3.5	3
9	M	36	R	3	2.5
10	F	38	L	2.5	3.5
11	F	44	R	4.5	3
12	M	59	R	12	6
Mean	/	47.8	/	4.90	2.37

### Examinations of clinical safety

There was no infection found in any patients after surgery. All patients had a stable occlusion as same as preoperative examination. The wounds of all patients healed well so that there were no serious postoperative scars.

There was no displacement, breakage, or loosening of the prosthesis components in postoperative CT at all follow-up points. Postoperative CT also showed that there were no any low density images between the prosthesis and host bone in all joints, and the masseter muscle attachment on the surface of the prosthesis and mandibular ramus was similar with the normal sides, but there was no lateral pterygoid muscle attachment on the head of the prosthesis in all operated joints.

The indices of the liver, and kidney function tests, blood, urine and stool analysis tests for all patients were within the normal range or with no clinical significance at all follow-up points.

### Examinations of clinical efficacy

#### Subjective assessment outcomes

The mean preoperative pain level was 7.17 ± 1.40, while the postoperative scores were 2.25 ± 1.71, 1.33 ± 1.30, 0.92 ± 0.90, and 0.67 ± 0.78 at 1, 3, 6, and 12 months postoperative follow-up points.

The mean score of the preoperative mandibular function was 6.00 ± 2.37, while, postoperatively, the scores were 3.83 ± 1.70, 2.92 ± 1.16, 2.25 ± 1.22, and 1.75 ± 1.29 at the respective follow-up points.

The mean preoperative subjective diet level was 5.83 ± 1.95 and the postoperative levels were 4.00 ± 1.95, 2.17 ± 0.72, 1.42 ± 0.79, and 1.17 ± 0.94 at the same follow-up points.

**Fig. 4:**
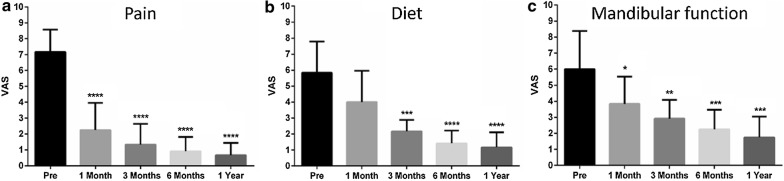
Subjective assessment outcomes over time. **a** Pain. **b** Diet. **c** Mandibular function

There were statistically significant improvements for pain, mandibular function, and diet at all postoperative follow-up intervals, except for the diet level at 1 month after surgery (P < 0.05) (Fig. 4).

#### Objective assessment outcomes

The mean preoperative MIO was 26.42 ± 9.30 mm and the postoperative values were 31.42 ± 7.62, 36.92 ± 6.16, 38.67 ± 6.08, and 39.25 ± 5.17 mm at 1, 3, 6 and 12 months after surgery. There were statistically significant improvements for MIO at 3, 6, and 12 months follow-up points (P < 0.01).

The mean preoperative MOD was 1.67 ± 1.37 mm with postoperative means of 4.71 ± 2.16, 3.83 ± 0.56, 4.13 ± 1.11, and 3.83 ± 0.98 mm. There was a statistically significant deviation to the normal side after surgery (P < 0.01).

The mean preoperative MDS was 4.79 ± 2.17 mm with postoperative means of 6.50 ± 1.88, 7.08 ± 1.82, 7.42 ± 1.69, and 7.50 ± 1.54 mm demonstrating statistically significant increases after surgery (P < 0.05). Regarding MNS, The mean preoperative value was 7.25 ± 2.21 mm, while the postoperative means were 3.13 ± 1.48, 2.96 ± 1.25, 2.92 ± 1.47, and 3.54 ± 1.10 mm revealing statistically significant decreases after surgery (P < 0.0001).

**Fig. 5:**
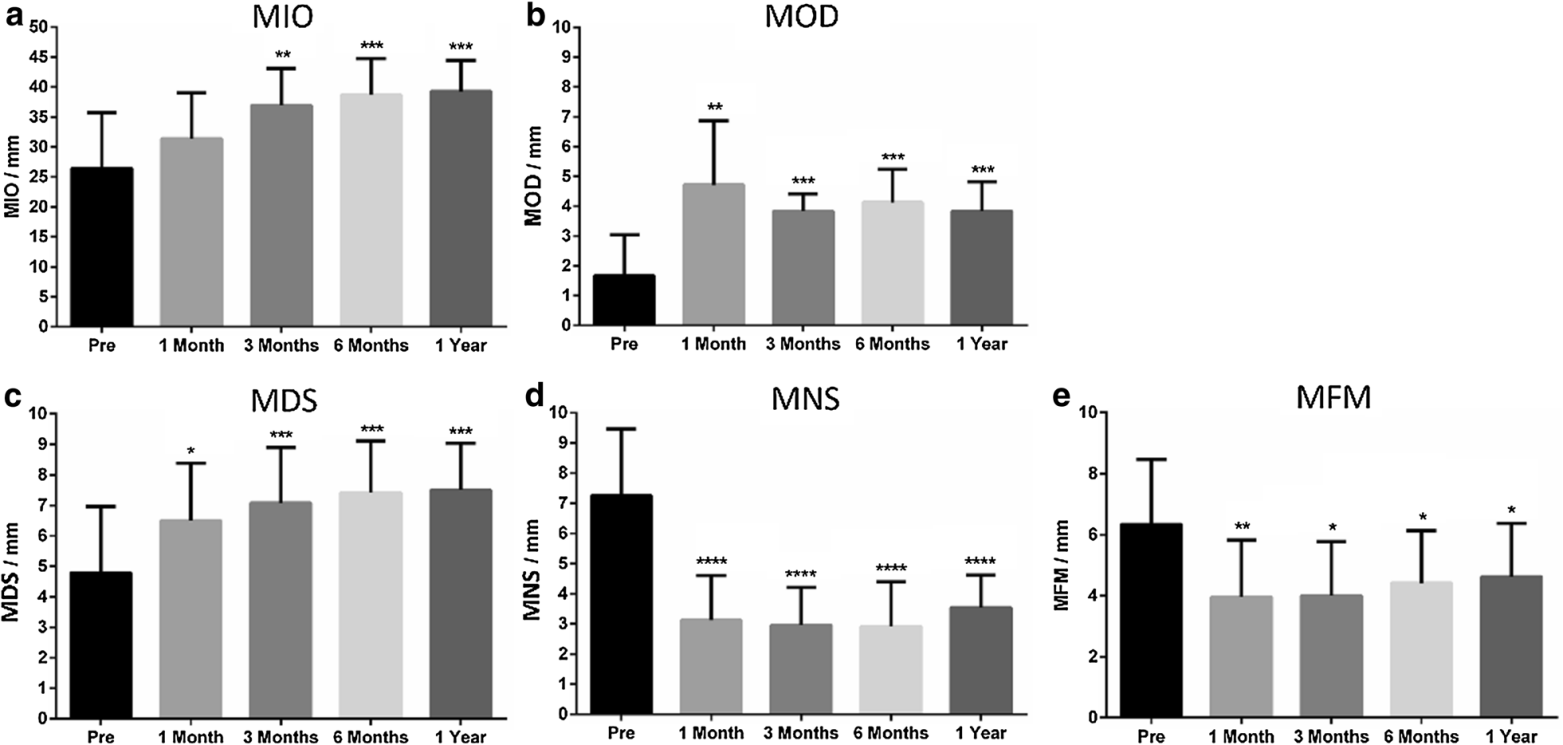
Objective assessment outcomes over time. **a** Maximal interincisal opening (MIO). **b** Mouth opening deviation (MOD). **c** Lateral movement to diseased side (MDS). **d** Lateral movement to normal side (MNS). **e** Mandible forward movement (MFM)

The mean preoperative MFM was 6.33 ± 2.14 mm with postoperative means of 3.96 ± 1.86, 4.00 ± 6.16, 4.42 ± 1.72, and 4.63 ± 1.75 mm at the corresponding follow-up points. There were statistically significant decreases for MFM after surgery (P < 0.05) (Fig. 5).

## Discussion

This study introduces a new TMJ prosthesis, which is totally different from the commercially available Zimmer Biomet and TMJ Concepts prostheses as for the design perspective, in addition to the manufacturing process. The clinical application of stock Zimmer Biomet is more common than the customized one. The stock Zimmer Biomet includes the fossa and mandibular components, in which the former is made of a grade of UHMWPE with a spherical articulating surface and a planar bony surface and the latter is fabricated with a single Co–Cr–Mo alloy accompanied by an oblate condylar head and planar mandibular handle (Fig. 1a) [10, 24]. TMJ Concept is a custom-made prosthesis, consisting of the fossa and mandibular components. The fossa is constructed of a pure titanium custom-made sheet with a welded mesh that interfaced with the dense UHMWPE articulating surface, and the mandibular component is constructed of 2 basic materials: Ti6Al4 V alloy coated with Co–Cr–Mo alloy head (Fig. 1b) [22, 23]. Particularly, the presented prosthesis in the current study is also a customized one which includes three parts: the fossa, condylar head, and mandibular handle components. The fossa component is the first innovative part of this prosthesis, which is a patient-specific design with single UHMWPE, which could match the anatomy of the fossa, zygomatic arch, and articular eminence very well. Moreover, the condylar head and mandibular handle components are constructed from Co–Cr–Mo and Ti6Al4 V alloys, respectively, which are connected together by the machine taper connection mechanism-a common connecting method for different metal materials in orthopedic prostheses (Fig. 1c). Based on the design principle, it is much easier and faster for the processes of manufacture and implantation in clinical application.

The 3D printing, as an additive manufacture method for the TMJ prosthesis, showed the second innovative point of this prosthesis, which is more consistent with the trend of medical development compared with the commercial Zimmer Biomet and TMJ Concepts prostheses manufactured by the conventional Casting Co–Cr–Mo alloy and wrought Ti6Al4 V alloys, respectively [10, 11, 22, 24]. As well known, a wide variety of 3D printing technologies have been advocated the medical field over the last three decades, especially for the fabrication of hip and knee joint prostheses in Orthopedic surgery [30]. Reviewing the kinds of literature, just one TMJ implantation device published in 2017 presented the use of 3D printing technique to fabricate the TMJ prosthesis for patients requiring joint replacement surgery [31, 32]. Its mandibular component with an oblate condylar head (same with TMJ Biomet) was fabricated from only titanium alloy by the 3D printing machine, while the condylar head of our prosthesis is constructed of Co–Cr–Mo alloy by 5-axis milling device, and only the mandibular handle component of our prosthesis constructed of titanium alloy by 3D printing. Theoretically, according to the development of the TMJ prosthesis, the functioning surfaces of TMJ prosthesis should have low wear, flow, and fatigue coefficients [9, 33, 34], therefore, the Co–Cr–Mo alloy has the prominent merits for use as the condylar head and may even show better outcomes in long-term follow-ups. In any case, both 3D printing prostheses have proved that modern 3D printing technology has enabled the more sophisticated, flexible, and automated production of TMJ prosthesis directly from CAD data.

The study is a prospective self-control research with very strict inclusion and exclusion criteria. Only patients diagnosed as unilateral end-stage TMJ osteoarthrosis in combination with a stable occlusion relationship have been recruited. Since John Murray Carnochan first reported an alloplastic TMJ reconstruction in the 1840s, most papers presented for TMJ prosthesis were retrospective researches [9, 13–15, 33, 34]. Limited literature is available addressing the clinical application of Zimmer Biomet or TMJ Concepts prostheses with the prospective design [10, 11, 22–24]. In addition, all these articles usually had a wide range of inclusion criteria, which included degenerated or resorbed joints, ankylosis, trauma, failed autogenous grafts, and other end-stage TMJ pathologies [10, 11, 22–24]. In fact, the wide inclusion criteria made the results offset, which eventually confuses the reader and also being difficult to understand and interpret the outcomes well for every specific type of TMJ diseases. As stated above, the results in the study would be more accurate and credible than before.

Clinically, in order to confirm the safety of the prosthesis in clinical use, we recorded the surgical complications, occlusion relationship, CT check after surgery, and some laboratory indices, including liver, and kidney function tests, routine blood, urine, and stool tests to determine if any related complications occurred postoperatively. From the comprehensive results (no severe maxillofacial complication occurrence, no other systematic organ damages, no displacement, breakage, and loosening of the prosthesis, and excellent bone contact with host bone), we realized that the safety of the prosthesis has been verified. However, Mercuri et al. reported the serum metal levels in patients who underwent different maxillofacial implanted metallic objects. The results showed the possibility of the increases of the metal levels including the cobalt, titanium, or chromium in the bloodstream after dental implant placement, orthognathic surgery using rigid metal fixation plates and screws, and total TMJ prosthesis. But they did not elucidate the clinical symptoms resulting from the metal level increases [35]. Nevertheless, we will also concentrate on the metal level analysis and possibly relative clinical discomforts for our patients in future follow-up to further confirm the safety of the TMJ prosthesis.

Moreover, completely postoperatively subjective and objective indices have been measured to confirm the efficacy of the prosthesis in 12 patients without missing case or in compliance for more than one year following surgery. The methods of follow-up, which have been widely used in previous studies for the clinical applications of Zimmer Biomet or TMJ Concepts products, were referred to the common criteria confirmed by Kent et al. in 1993 [22, 23, 33]. Based on these criteria or method, our study showed an average of 90.7% decrease in pain, 70.8% improvement in mandible function, 79.9% improvement in diet, and 32.8% increase in MIO at 1 year after surgery. From 1993 to 2017, there were many studies that evaluated the postoperative efficacy of TMJ prostheses (mainly TMJ Biomet and Concepts) using the same criteria. Their results showed 48–78.1% decrease in pain, 51–60% improvement in mandible function, 51.5–69.5% improvement in diet, and 23.9–66% increase in MIO [10, 11, 22–24]. The improvements in pain, mandible function, and diet in our study were more obvious compared to other studies. This could be related to the inclusion criteria. In our study, patients with TMJ osteoarthrosis were included only, usually presenting with periarticular pain. Meanwhile, the mandible functions, diet, and MIO have been limited due to pain. In other studies, the included patients were usually recorded with different types of TMJ pathologies (osteoarthritis, ankylosis, idiopathic condylar resorption, and so on) together, so that the evaluated indices have been influenced by each other. For lateral and forward movements, and opening mouth deviation, we found some negative outcomes, including the significant limitations of the mandible forward movement and lateral movement to the normal side, and the deviation to the operated side when mouth opening. These were attributed to the attachment loss of the lateral pterygoid muscle, which usually helps the mandible move forward and contralaterally. Meanwhile, the finding of the attachment loss between this muscle and the prosthesis in postoperative CT images can further verify this reason. Actually, these negative results have been found and explained in previous studies [11, 23]. But they just found the limitation to the contralateral joint side. There were no self-control results to show how serious the problems were, and no postoperative CT to check the attachment of the muscle. Therefore, the evidence in this study was more persuasive because of being prospectively self-control project, where only unilaterally operated patients were included. As a result, the efficacy of the new prosthesis has been confirmed based on the subjective and objective indices.

## Conclusions

The presented TMJ prosthesis is an innovative product in TMJ Yang’s system due to its special design, 3D printing additive fabrication, and corresponding surgical procedure with the help of surgical templates and endoscopy. Moreover, this prospective self-control study proved the safety and efficacy of the prosthesis by clinical, radiological and laboratory examinations and comparisons in detail. This study will support the evidence for an extensive clinical application of the innovative prosthesis in the future.

## Abbreviations

TMJ: temporomandibular joint
CT: computed tomography
CAD/CAM: computer-aided design and computer-aided manufacturing
3D: three-dimensional
UHMWP: ultra-high-molecular-weight polyethylene
Co–Cr–Mo: cobalt-chromium-molybdenum
VAS: visual analog scale
MIO: maximal interincisal opening
MDS: movement to diseased (operated) side
MNS: movement to normal (non-operated) side
MFM: mandible forward movement
MOD: mouth opening deviation
